# Characterization of the effects of vitamin D synthesis and sunburn in the population due to solar radiation exposure using PROBIT methodology

**DOI:** 10.1016/j.heliyon.2024.e30864

**Published:** 2024-05-11

**Authors:** Juan Francisco Sánchez-Pérez, Begoña Comendador-Jiménez, Enrique Castro, Manuel Cánovas, Manuel Conesa

**Affiliations:** a Department of Applied Physics and Naval Technology, Universidad Politécnica de Cartagena, Spain; b General Directorate of Pharmacy and Health Products. Conselleria de Sanidad Universal y Salud Pública. Comunitat Valenciana. Spain; c Department of Metallurgical and Mining Engineering, Universidad Católica del Norte, Chile

**Keywords:** Ultraviolet index, Vitamin D generation, Degree of sunburns, PROBIT methodology, Education of the population

## Abstract

The main objective of this work is to present a set of equations that relates exposure time to solar radiation, the ultraviolet index (UVI), and its effects, both positive (vitamin D synthesis) and negative (sunburns), including the influence of repeated doses and the incorporation of protective factors. To do so, expressions are proposed for both effects and a time range is specified where repeated doses can be applied. Moreover, we propose expressions (PROBIT equations) that relate the percentage of a population that would reach the recommended daily amount of vitamin D and the repeated dosage to which the population is exposed for skin types I to IV. For all the cases studied, protective factors, such as the use of clothes or sunscreens, were taken into account. Additionally, comparisons were made based on skin types between the generation of daily vitamin D and the onset of sunburn, being able to establish a percentage of those who would suffer from first-degree sunburns when this population reached the recommended daily amount of vitamin D. Thus, it can be noted that when a large percentage of a population wants to obtain the recommended daily allowance of vitamin D of 2000 IU or more, and by exposing a small portion of skin to solar radiation, a considerable percentage of that population would suffer from first-degree sunburn as vitamin D generation is related to the area of exposed skin. Therefore, by increasing the area of skin that is exposed to solar radiation, vitamin D needs can be safely met even with higher daily amounts.

## 1 Introduction

Currently, in some cultures, tanned skin is associated with well-being and health, which is why people overexpose themselves to the sun's rays in order to get a tan [1,2]. This leads to two completely opposite results: on the one hand, an increase in the incidence of skin cancer or sunburn due to overexposure to the sun, and on the other hand, the necessary vitamin D dose is reached [3].

Earth receives radiation in the electromagnetic spectrum and is classified into bands according to the frequency and wavelength. Inside this spectrum is solar radiation: infrared (heat sensation), visible light (retinal stimuli), and ultraviolet radiation (UVR) A, B, and C, which are on the health spectrum, are of great interest [4]. The total UVR that reaches the population is the sum of direct radiation, passing in a straight line from the sun through the atmosphere, while diffuse radiation is dispersed by the atmosphere; and reflected UVR, bounces on surfaces and later impacts on people's skin. UVR produces different alterations to the skin; it can be beneficial in the synthesis of vitamin D and causes acute harm in the form of sunburn, which can lead to chronic damage such as skin cancer. For example, recent studies have shown that low doses of solar radiation in summer produce sufficient vitamin D and low-level non-cumulative DNA damage in light-skinned individuals. However, this same dose produces less vitamin D and less DNA damage in brown-skinned individuals [5]. Other authors have indicated that this basal damage to cells that occurs at low levels of ultraviolet radiation explains why light-skinned individuals have a higher incidence of skin cancer [6].

Thus, the literature indicates that solar radiation mainly produces first-degree sunburns, with second-degree sunburns being less frequent [[7], [8], [9], [10], [11], [12]]. The main effect of the former is reddening of the skin, making it more sensitive and causing pain and swelling (erythema). The second is characterized by blister formation and more intense pain [7,8]. Consequently, this study focuses mainly on first-degree burns.

In the strictest sense, vitamin D is an endocrine hormone since our body is able to synthesize it. There are two sources: one is from foods such as oily fish, eggs, or milk, but the main source (>90 %) is synthesized in the skin by exposure to the sun [13]. After exposure to sunlight, 7-dehydrocholesterol in the skin absorbs UVB radiation and is converted to previtamin D_3_, which in turn is isomerized to vitamin D_3_. Vitamin D is first metabolized in the liver to its main circulating form in the liver, 25-hydroxyvitamin D, and then in the kidneys to its biologically active form, 1,25-dihydroxyvitamin D, which plays an essential role in the maintenance of skeletal health and metabolic functions through the regulation of calcium and phosphate metabolism [14,15]. In addition, it should be noted that its synthesis is influenced by the time of year, altitude, time of day, skin pigmentation, and use of sunscreens, among others, in fact, sunscreens block the formation of previtamin D_3_, if properly applied [16,17].

There is ample evidence that supports the importance of vitamin D in the functioning of the organism, such as the regulation of calcium and phosphorus, the metabolism of insulin, regulation of the immune system, cardiovascular and skeletal muscle systems, etc. [18]. There are studies that postulate an association between the presence of vitamin D and an improvement in the recovery of skin lesions, being able to act as a treatment for acute cutaneous lesions induced by UVR [19,20], inflammation attenuation and pain reduction. Consequently, according to previous studies, vitamin D deficiency may put a person at risk of suffering from rickets, osteoporosis, breast cancer, multiple sclerosis, diabetes mellitus, and other aliments [21].

Regarding the recommended amount of vitamin D_3_, several studies have established doses between 400 and 1000 International Unit (IU), or the same as, 10–25 μg of vitamin D_3_. However, some treatments have indicated doses between 2000 and 4000 IUs per day for a period of 3–6 months, or the same as 50–100 μg of vitamin D_3_ [14,[22], [23], [24], [25]]. Regarding the recommended amount depending on age, the Endocrine Society concluded in a study that for children aged 0–1 years, an amount of vitamin D between 400 and 1000 IU would be recommended, for ages between 1 and 18 years, between 600 and 1000 IU, and finally, for adults, between 1500 and 2000 IU of vitamin D per day [15,26]. Finally, regarding the time required for the population to be exposed to solar radiation to reach the recommended amount of vitamin D, several studies have related this time to ultraviolet index (UVI) and skin type [22,27,28].

Consequently, the importance of UVR in humans has been demonstrated, but its harmful effects must not be forgotten. UVA radiation, also known as "aging radiation,” causes damage to the dermis, which is related to photoaging, destruction of fibroblasts, and the appearance of oxidative stress. UVB radiation, known as "burn radiation,” causes damage mainly to the epidermis and has been related to sunburn and DNA damage. These lesions are repaired automatically by cellular mechanisms. However, a cumulative effect of these lesions can lead to the organism being unable to repair them, triggering the onset of a skin neoplastic process [29].

The risk associated with sunburn throughout one's life is associated with cutaneous melanoma. Studies have shown that the greater the number of sunburns suffered throughout one's life, the greater the probability of acquiring cancer [30]. In addition, there is a relationship between the appearance of skin cancer and episodes of severe burns during the 5 years prior to the onset of the disease [31]. In a monograph published by the International Agency for Research on Cancer (IARC) on the carcinogenic risk in humans due to radiation, there was a significant association between carcinoma and cumulative exposure to the sun and episodes of multiple sunburns [32].

As a key aspect, Finney in 1971 [33] proposed the PROBIT (PROBability unIT) methodology as a simplified way to quantify the effect or damage to populations through the PROBIT function by relating it to the variable that causes the effect or damage. In this study, based on this PROBIT methodology, we have obtained a set of equations that allows us to relate ultraviolet radiation, and the variables that cause the effect or damage, with the percentage of the population that would suffer it. This method has been used to quantify the effects or damage caused by almost any hazardous agent, such as blast overpressure, toxic gas and so on [[34], [35], [36], [37]].

## 2 Methodology for developing the model to determine the effects caused by solar radiation

### 2.1 Ultraviolet index, dose, and intensity

The UVI, developed by scientists in the Environment and Climate Change of Canada and adopted in the international program of the United Nations Environment Program and World Health Organization (WHO), establishes a simple risk relationship between UVR and its danger according to the Commission Internationale de l’Eclairage, which establishes the reference action spectrum for UV-induced erythema on the human skin. Thus, for low values (less than or equal to five), there is a low or moderate risk. For larger values, the range varies from high to extreme [[37], [38], [39], [40]]. On the other hand, this Commission also proposed a technical report on the action spectrum for the production of previtamin D_3_, which is still under study [41,42].

This index, UVI, encompasses the entire spectrum of UVR, that is, UVC including a wavelength between 100 and 280 nm, UVB, between 280 and 315 nm, and finally, UVA, between 315 nm and 400 nm [39].

When solar radiation passes through the atmosphere, all UVC radiation and 90 % of UVB radiation are absorbed by ozone, water vapor, oxygen, and carbon dioxide. UVA radiation is less affected by the atmosphere. Therefore, the ultraviolet radiation that reaches Earth's surface is composed mainly of UVA radiation and a minor part of UVB radiation.

Thus, UVI encompasses the spectrum between 250 nm and 400 nm and can be calculated by integrating the wavelength range with the product of the solar spectral irradiance and erythema reference action spectrum coefficient (*ε*(λ)) multiplied by a constant with a value of 40 m^2^/W. Moreover, factors to be taken into account are the sun position, latitude, cloudiness, altitude, ozone and ground reflection [39]. The UVI is accessible on numerous meteorological data websites, such as the website of the Finnish Meteorological Institute [43]. The erythema reference action spectrum coefficient is a function of wavelength [38,39,44].

The dose is a variable used in numerous situations where there is an agent that can cause damage or ill effects, such as solar radiation, vibrations, noise, the concentration of pollutants and so on, to establish a threshold at which damage or ill effects occur. In all cases, the dose is made up of two factors: the concentration or amount of the agent that causes the damage and the exposure time (t_e_, s). For several kind of radiation, the damage-causing variable is the intensity (I, W/m^2^). Thus, the dose (D, (kW/m^2^)^4/3^ s) can be expressed as shown in equation (1) [37,45,46].

(1) $$D=I^{4/3}t_{e}\;{\left(\text{kW}/m^{2}\right)}^{4/3}\;s$$

where the intensity exponent can take a value of 1.15 or 4/3, depending on the type of radiation or damage [46]. In other works, on the expression for the calculation of the erythematous dose, the exponent mentioned above seems to take the value of unity, thus using a linear relationship to calculate the dose, and from this it is possible to establish the exposure time necessary to produce erythema on the skin. Currently, an expression used for the calculation of the sun exposure time necessary to induce erythema would be equation (2) [22,[47], [48], [49], [50]]:

(2) $$t_{e}=\frac{40\;\left(m²/w\right)}{60\;\left(s/\min\right)}\frac{\text{MED}\;\left(J/m²\right)}{\text{UVI}}\min$$

where UVI = 40 ·UV_Ery_, being UV_Ery_ (W/m^2^) erythemally weighted UV radiation and 40 (m^2^/W) the constant mentioned above. This expression is in accordance with the data given by the guide for publication and interpretation of solar UV Index forecasts for the public prepared by the Working Group 4 of the COST-713 Action (European Cooperation in Science and Technology) and by DIN-5050 which relates the UVI and 1 Minimum Erythema Dose (MED) for skin types I to IV [[47], [48], [49]].

Thus, from expression (2) we can obtain equation (3) for the dose, that when its value coincides with the MED would induce erythema, Table 1.

(3) $$D=\frac{60\;\left(s/\min\right)}{40\;\left(m²/w\right)}\text{UVI}\;t_{e}\;\left(\min\right)\;J/m^{2}$$

**Table 1:** MED for erythema for several skin type [22,51,52].

Skin type classification	MED (J/m^2^)
I	200
II	250
III	350
IV	450

### 2.2 Vitamin D and sunburn

**Fig. 1:**
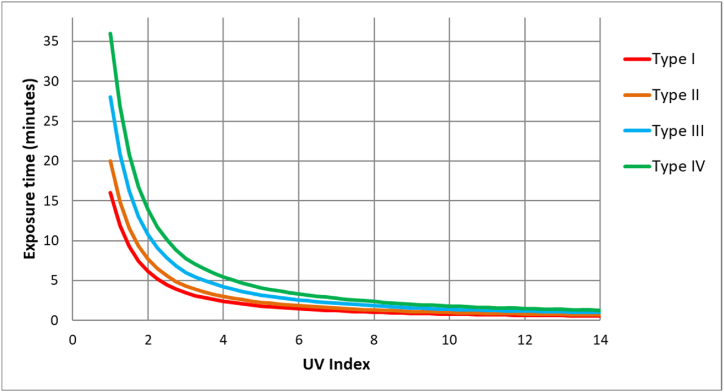
Relationship between the exposure time necessary for 97 % of the population to reach the daily necessary vitamin D amount of 1000 IU and UV index on clear days for several skin types and full body exposure. Built with the expression developed by McKenzie et al. and the data that relate the ratio UV_VitD_ ⁄UV_Ery_ and UVI [22].

As indicated above, several authors recommend doses of vitamin D_3_ between 400 and 4000 IU; therefore, 400, 1,000, 2,000, and 4000 IU of vitamin D will be studied in this paper [16]. McKenzie et al. [22] propose a relationship to determine the minimum exposure time required for the population to reach the daily vitamin D intake of 1000 IU for various skin types [22,39]. After reviewing the information provided by McKenzie et al. and by Webb and Engelsen [22,27], it can be assumed that practically the whole population would reach 1000 IU of vitamin D after a certain time exposed to the corresponding solar radiation, so it can be assumed that approximately 97 % of the population would reach the daily recommended amount of 1000 IU based on the Recommended Dietary Allowance [53]. Finally, Fig. 1, constructed using the expression developed by McKenzie et al. [22] and data relating the ratio of UV_VitD_ ⁄UV_Ery_ and UVI, shows the relationship between the exposure time required for 97 % of the population to reach the daily necessary vitamin D_3_ amount of 1000 IU and the UV index on clear days for various skin types and full body exposure.

The choice of skin types I to IV is due to the fact that skin types V and VI rarely suffer from sunburn, and on the other hand, as mentioned above, that the time needed to reach the recommended dose of vitamin D is between 5 and 10 times that needed for skin type II, and it is not clear which factor should be applied [22,54].

The expression of McKenzie et al. mentioned above for calculating the time needed to reach the recommended amount of vitamin D for a fully exposed person without sun protection can be rewritten as shown in equation (4) [22]:

(4) $$t_{e,\text{vit}\;D}=\frac{1}{k\;R_{\text{VitD}/\text{Ery}}}\frac{\text{MED}\;}{\text{UVI}}\;\min$$

where k is a constant that includes geometrical, biochemical and physiological considerations and R_VitD/Ery_ is the ratio UV_VitD_ ⁄UV_Ery_, being UV_VitD_ the vitamin D-weighted UV radiation. Knowing that the time needed to reach a vitamin D dose of 1000 IU for a skin type II person fully exposed to a solar radiation of UVI 10 (peak UV for mid-latitudes in the northern hemisphere) and R_VitD/Ery_ value of 2 is approximately 1 min, a value of 12.5 (J/m^2^ min) for k is obtained [22]. This constant k has the same units as the 60/40 ratio of equation (3). Analogously to expression (3) an equation for the vitamin D dose can be obtained, equation (5), where by including the correcting factors k and R_VitD/Ery_ (ratio UV_VitD_ ⁄UV_Ery_) the dose at which the recommended value of 1000 IU is reached can be assumed to be the MED, since the constant k has been adjusted using the MED as a threshold value to reach the aforementioned recommended value [22]. The value of the dose required to reach the recommended amount of 400, 2000 and 4000 IU shall be calculated as a ratio to the value required for 1000 IU.

(5) $$D_{\text{Vit}\;D}=k\;R_{\text{VitD}/\text{Ery}}\;\text{UVI}\;t_{e,\text{vit}\;D}\;J/m^{2}$$

On the other hand, and derived from the above, expression (3) could also be used including the R_VitD/Ery_ factor, expression (5′), but establishing new threshold values for which 97 % of the population could reach the amount of 1000 IU of vitamin D_3_, Table 2. It should be noted that both expressions (5) and (5′) would provide the same times to reach the recommended dose of 1000 IU, since the coefficients and threshold values of both expressions are proportional. As can be seen, equations (3), (5) are the same expression where the R_VitD/Ery_ coefficient takes, by its own definition, a unity value in the case that the effect produced by solar radiation is erythema.

$$D_{\text{Vit}\;D}=\frac{60\;\left(s/\min\right)}{40\;\left(m²/w\right)}R_{\text{VitD}/\text{Ery}}\;\text{UVI}\;t_{e,\text{vit}\;D}\;J/m^{2}\;\left(5’\right)$$

**Table 2:** Minimum dose value for an amount of 1000 IU of vitamin D_3_ (MVD) for several skin type and for expression (5’).

Skin type classification	MVD (J/m^2^)
I	24
II	30
III	42
IV	54

**Fig. 2:**
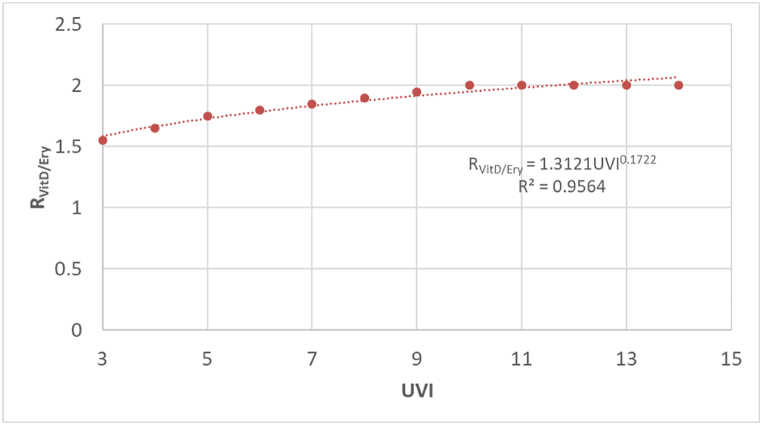
Adjustment of the relationship of the ratio UV_VitD_ ⁄UV_Ery_ with the UVI [22].

On the other hand, the R_VitD/Ery_ value has been adjusted from the data given by McKenzie et al. for an interval for a UVI of 3–14, equation (6) (Fig. 2).

(6) $$R_{\text{VitD}/\text{Ery}}=1.3121\;{\text{UVI}}^{0.1722}$$

Finally, combining expressions (5), or expression (5’), and (6) and the value calculated above for k gives the expression for the dose of vitamin D, equation (7) or (7′), respectively.

(7) $$D_{\text{Vit}\;D}=16.40\;{\text{UVI}}^{1.1722}\;t_{e,\text{vit}\;D}\;J/m^{2}$$

$$D_{\text{Vit}\;D}=1.97\;{\text{UVI}}^{1.1722}\;t_{e,\text{vit}\;D}\;J/m^{2}\;\left(7^{\prime }\right)$$

### 2.3 Repeated doses and protection factor

The initial dose (D, J/m^2^) for both the production of vitamin D_3_ and sunburn can be achieved by repeated exposure to UVR. In other words, the population may be exposed to a different IUV during the day or days.

For vitamin D_3_, the population must reach the required daily amount of 400 IU, 1000 IU, 2000 IU, or 4000 IU with repeated doses. During this time, a population can be exposed to solar radiation reaching the recommended value, but clothing or sunscreens that block or attenuate solar radiation on their skin can be used, thus decreasing the effective dose (D, J/m^2^) received. Thus, Equations (3), (7), or expression (7′), are modified to quantify this effect, equations (8), (9) or (9’), respectively:

(8) $$D=\sum_{1}^{n}D_{i}\frac{\text{AF}}{\text{SPF}}=\sum_{1}^{n}\frac{60\;}{40\;}{\text{UVI}}_{i}\;t_{e,i}\frac{1}{\text{SPF}}\;J/m^{2}$$

(9) $$D_{\text{Vit}\;D}=\sum_{1}^{n}D_{\text{Vit}\;D,i}\frac{\text{AF}}{\text{SPF}}=\sum_{1}^{n}16.40\;{\text{UVI}}_{i}^{1.1722}{\;t}_{e,\text{Vit}\;D,i}\frac{\text{AF}}{\text{SPF}}\;J/m^{2}$$

$$D_{\text{Vit}\;D}=\sum_{1}^{n}D_{\text{Vit}\;D,i}\frac{\text{AF}}{\text{SPF}}=\sum_{1}^{n}1.97\;{\text{UVI}}_{i}^{1.1722}{\;t}_{e,\text{Vit}\;D,i}\frac{\text{AF}}{\text{SPF}}\;J/m^{2}\;\left(9’\right)$$

where n is the number of exposures to the Sun, AF is the area factor, and SPF is the sun protection factor. The AF, expressed in parts per unit, takes the unit value when the entire body is exposed to solar radiation. It should be noted that the area factor does not appear in the expression (8) for sunburn, since unlike the production of vitamin D, where increasing the exposed area can increase the aforementioned production, in the case of sunburn, localized burns may occur depending on the area exposed to solar radiation. The approximate AF values for the different body parts are listed in the following table (Table 3) [22].

**Table 3:** Area factor (AF) for different body parts.

Body part exposed to the Sun	AF
Face	0.09
Face and hands	0.10
Face, hands and arms	0.27
Face, hands, arms and legs	0.63
Face, hands, arms, legs, back and chest	0.92
Full body	1.00

### 2.4 PROBIT equations to determine vitamin D synthesis and sunburn for repeated doses

A key aspect in calculating the harmful effects of sun exposure is the work of Finney in 1971 [33]. The PROBIT methodology was proposed as a simplified method to quantify the effect or damage to populations through the PROBIT function or equation by relating it to the variables that cause the effect or damage. This method has been used to quantify the effect or damage caused by almost any hazardous agent, such as blast overpressure effects, toxic gas and so on [[34], [35], [36], [37]].

By adjusting the values given in Table 1, or Table 2, depending on whether expression (9) or (9′) is used, the PROBIT equations for skin types I to IV can be established so that 97 % of the population would reach the recommended vitamin D_3_ requirement of 400, 1,000, 2000 and 4000 IU, respectively, equation (10) [37,45]:

Equations for Vitamin D generation and skin types I to IV

(10) $$Y=\alpha +6.95\;\log\left(D_{\text{Vit}\;D}\right)$$

where Y is the PROBIT value**.** Table 4 presents the value of the α coefficient of equation (10) for skin types I to IV and vitamin D value for both equations (9), (9). Obviously, and derived from what was explained above, both expressions provide the same PROBIT values (Y).

**Table 4:** α coefficient value of equation (10) for skin types I to IV and vitamin D value.

Vitamin D (IU)	α value							
	Type I		Type II		Type III		Type IV	
	Eq. (9)	Eq. (9’)	Eq. (9)	Eq. (9’)	Eq. (9)	Eq. (9’)	Eq. (9)	Eq. (9’)
400	−6.35	0.05	−7.02	−0.62	−8.04	−1.64	−8.79	−2.39
1000	−9.11	−2.71	−9.79	−3.39	−10.80	−4.40	−11.56	−5.16
2000	−11.20	−4.80	−11.88	−5.48	−12.89	−6.49	−13.65	−7.25
4000	−13.30	−6.90	−13.97	−7.57	−14.99	−8.59	−15.74	−9.34

These equations have been corroborated on the one hand, knowing that the time necessary for a person with skin type II with an area factor of 1 to reach 1000 IU of vitamin D at a UVI of 10 is approximately 1 min [22], and on the other hand, with the data given by Webb and Engelsen [27] for Boston, taking an area factor of 0.27 for the end of March and June and a mean value of UVI at local solar noon and a clear day [55]. An example of the application of the proposed equations to Boston data given by Webb and Engelsen is presented in Section 3 [27]. Similarly, using Table 1, PROBIT equations for first-degree sunburn for skin types I-IV can be established for 1 % of the affected population, (equation (11) and Table 5):

**Table 5:** β coefficient value of equation (11) for skin types I to IV and sunburn degree.

Sunburns	β value			
	Type I	Type II	Type III	Type IV
first-degree	−13.32	−14.00	−15.01	−15.77

Equations for sunburns and skin types I to IV

(11) Y=β+6.95log(D)

It is worth noting that first-degree sunburns have been studied, as they are the main type of sunburns that occur after sun exposure [[7], [8], [9], [10], [11], [12],^56^].

**Fig. 3:**
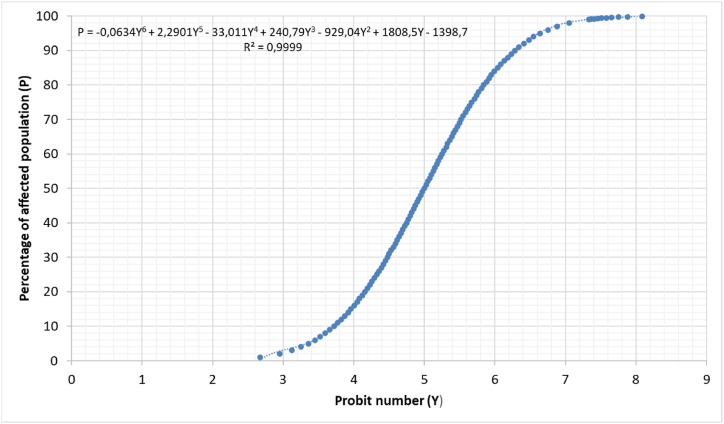
PROBIT table adjustment.

The equivalence between Y and the percentage of the affected population is made with the table given by Finney [33]. In this study, we adjusted the values of the PROBIT table to the following expression (12) that relates Y to the percentage of the population affected (Fig. 3):

(12) $$P=‐0.0634Y^{6}+2.2901Y^{5}‐33.011Y^{4}+240.79Y^{3}‐929.04Y^{2}+1808.5Y‐1398.7$$

where P is the affected population percentage and the expression is valid for the range of Y between 2.67 and 8.09. For Y values lower than 2.67 the affected population percentage is zero.

**Fig. 4:**
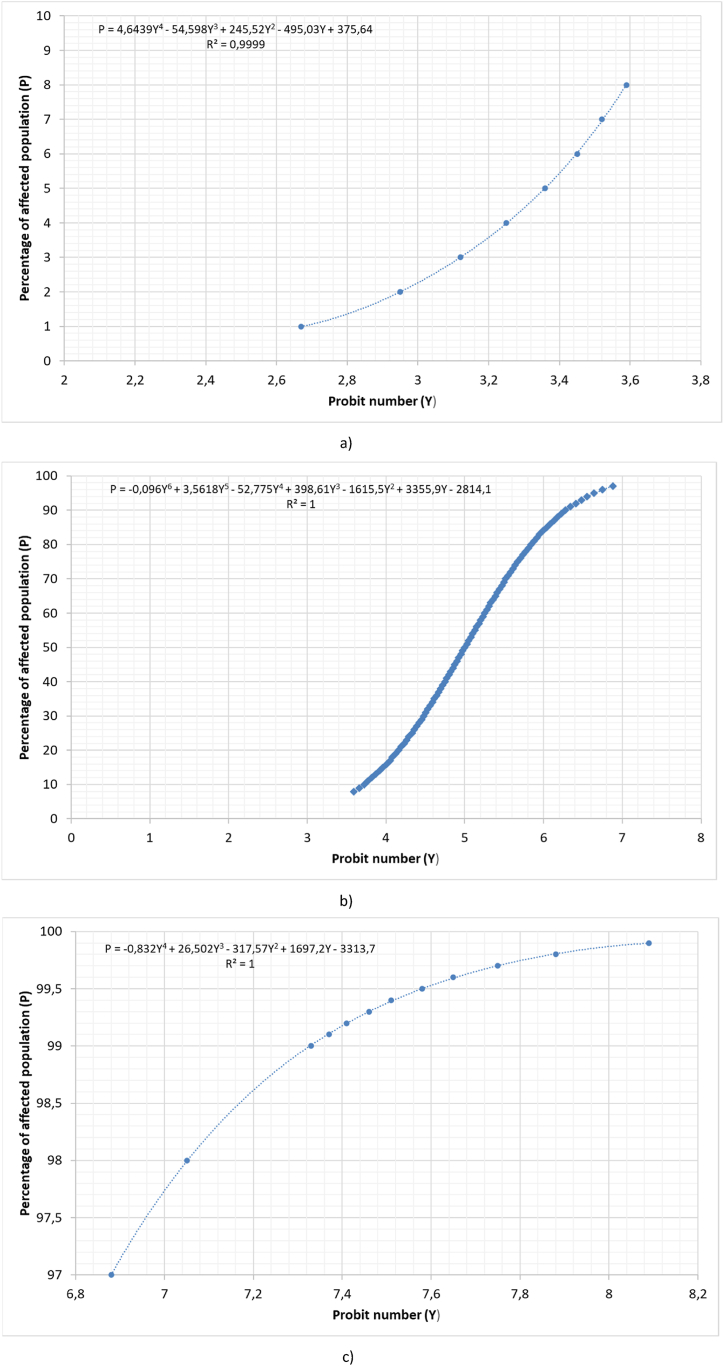
PROBIT table adjustment for Y range between a) 2.67 and 3.59, b) 3.72 and 6.9 and c) 6.9 and 8.09.

If we look at Fig. 3, we can see that the previous expression is imprecise at the extremes; therefore, three expressions are proposed that cover the entire PROBIT table with greater precision (equation (13) to (15) and Fig. 4(a–c)):

(13) $$2.67\leq Y\leq 3.59P=4.6439Y^{4}‐54.598Y^{3}+245.52Y^{2}‐495.03Y+375.64$$

(14) $$3.59\leq Y\leq 6.9P=‐0,096Y^{6}+3.5618Y^{5}‐52.775Y^{4}+398.61Y^{3}‐1615.5Y^{2}+3355.9Y‐2814.1$$

(15) $$6.9\leq Y\leq 8.09P=‐0.832Y^{4}+26.502Y^{3}‐317.57^{2}+1697.2Y‐3313.7$$

## 3 Results, discussion and study cases: relationships between the percentage of a population affected by solar effects and UVI

Next, the synthesis of vitamin D was studied for cases of 400, 1,000, 2,000, and 4000 IU and its relationship with first-degree sunburns. In all cases, the figures show the relationship between the time required for each of the damages (sunburns) or benefits (synthesis of vitamin D) for UVI values between 3 and 12. The choice of these values is due to the fact that for lower values, a longer time would be needed or vitamin D synthesis would not occur. It should be noted that the use of UVI in this paper is because it is a standardized and widely used index that encompasses factors that influence the intensity of UV radiation at ground level such as wavelength, sun position, latitude, cloudiness, altitude, or ozone [39]. These factors can influence by decreasing the intensity, as occurs with cloudiness. An example of its calculation is shown in "Learn About the UV Index" by U.S. Environmental Protection Agency (US EPA) [57]. Finally, since equations (9), (9) with their corresponding α coefficients for expression (10) provide the same PROBIT (Y) values, only equation (9) with its corresponding α values will be used.

**Fig. 5:**
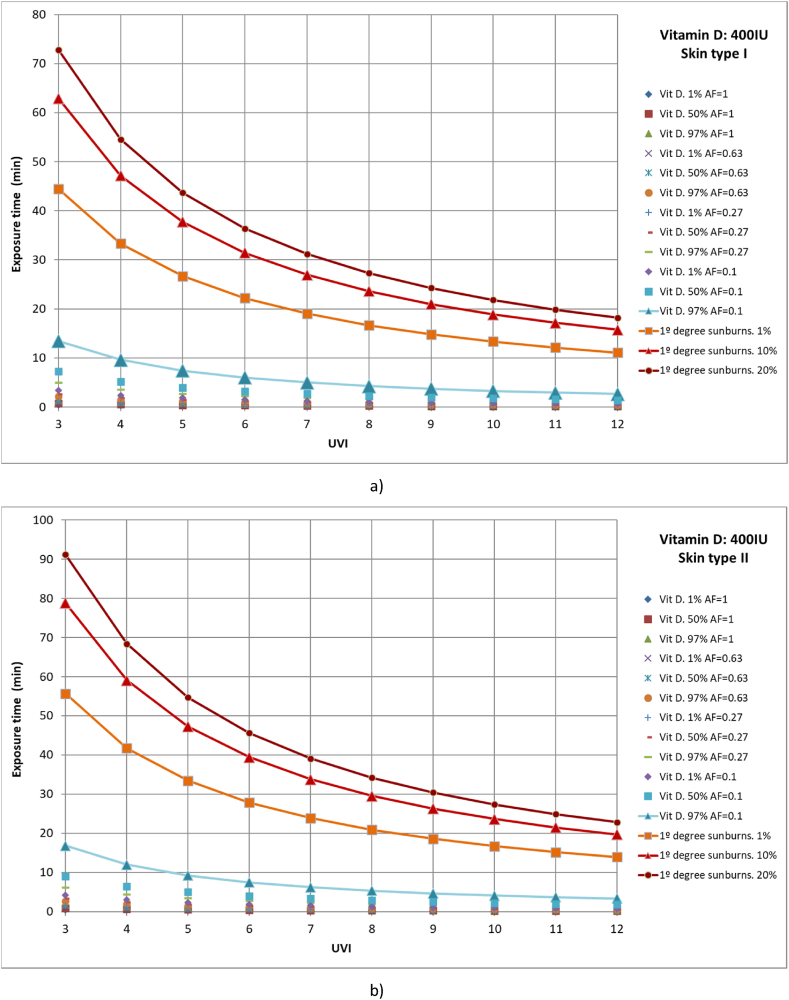

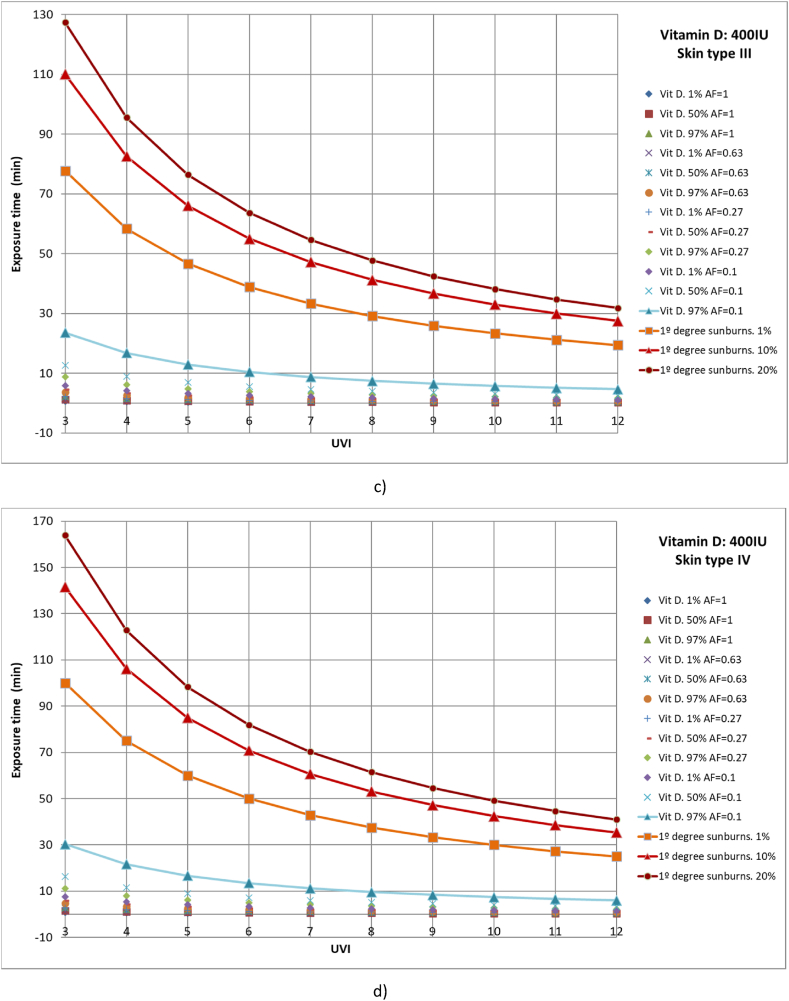
Representation of exposure time and UVI for the generation of vitamin D of 400IU and first-degree sunburns for different affected population percentages of a) skin type I, b) skin type II, c) skin type III and d) skin type IV.

First, 1 %, 50 %, and 97 % of the population with different body parts exposed to solar radiation who reach a vitamin D generation of 400 IU was studied. For example, to calculate the time required for 50 % of the population with skin type I that only exposes the face and hands to the sun (AF = 0.1) to reach 400 IU of vitamin D and a UVI of 8, the first Y must be obtained from equation (12) or (14) or from Finney's PROBIT Table [33], taking a value of Y = 5. Then, from Equation (10), the dose required for this case can be obtained, taking a value of 43 J/m^2^. Finally, with expression (9), the value of the time, t_e_ ≈ 2.3 min, can be obtained. We analyzed the cases of full body exposure to solar radiation; exposure of the face, hands, arms, and legs; exposure of the face, hands, and arms; and finally, face and hands (Fig. 5(a–d)). In addition, the results were compared with those of 1 %, 10 %, and 20 % of the population affected by first-degree sunburns. It should be noted that the rate of vitamin D synthesis is directly related to the area of exposed skin; however, sunburns do not have this relationship, being able to produce localized burns in the parts exposed to solar radiation (Fig. 5(a–d)).

Across all skin types studied and area factors, 97 % of the population reached the recommended daily allowance of vitamin D of 400 IU without first-degree sunburn. Analyzing first-degree burns, as the UVI increases, the time is considerably reduced, approaching the time required for virtually the entire population to reach the necessary vitamin D level when only the hands and face are exposed.

**Fig. 6:**
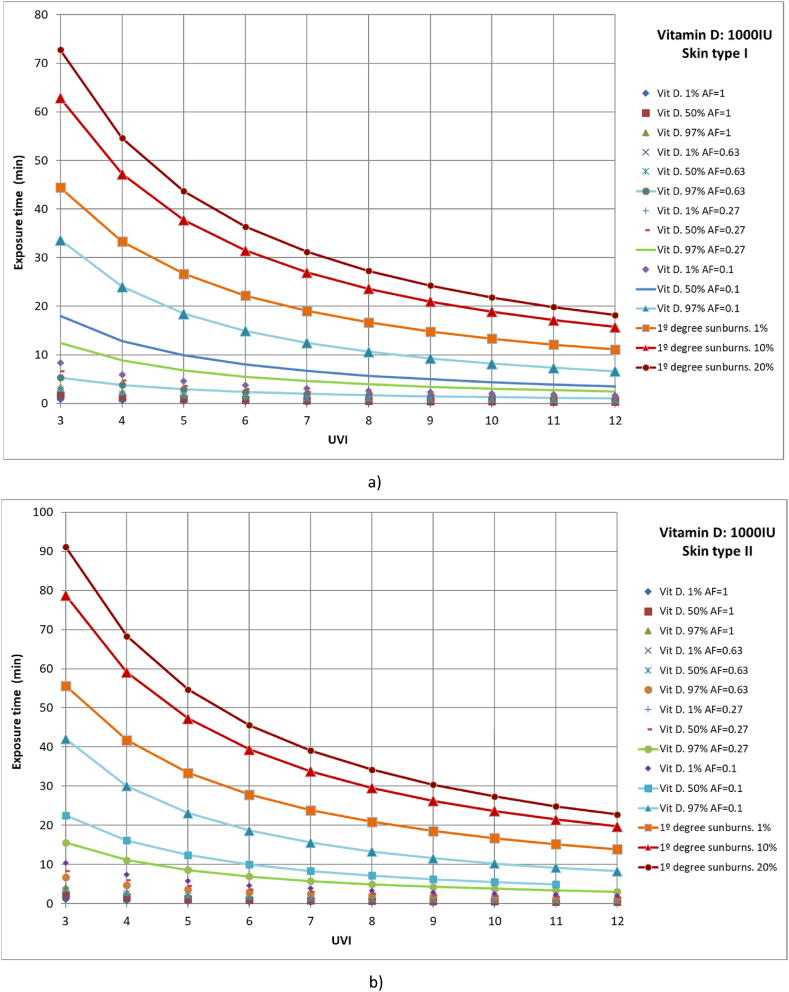

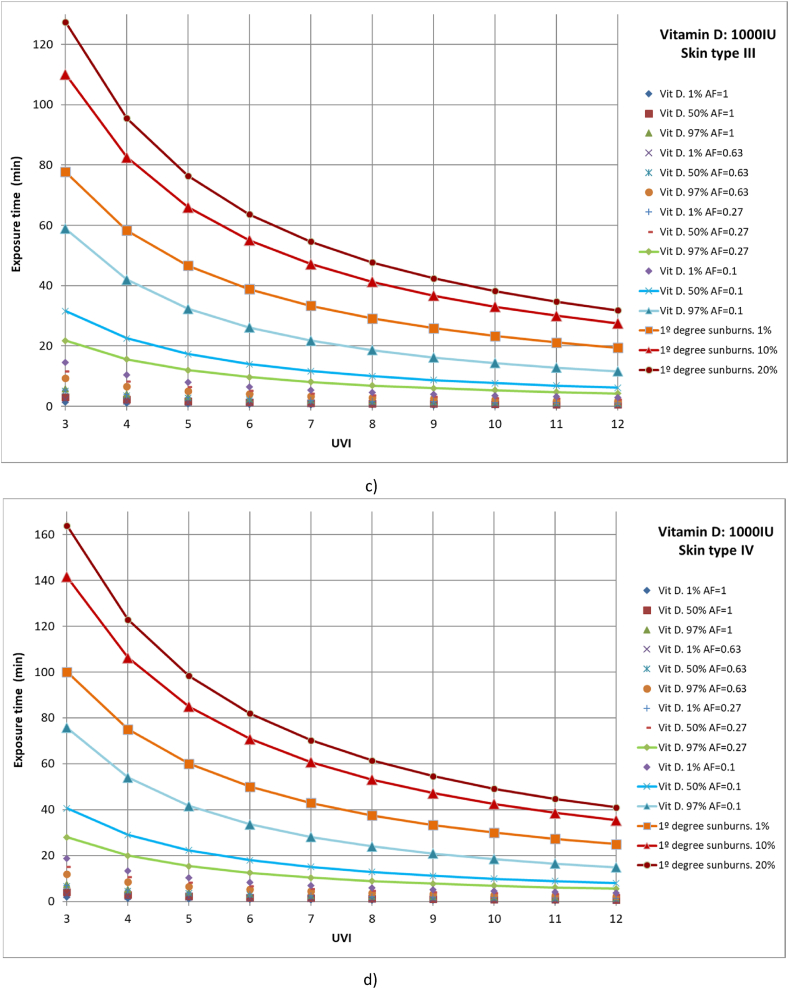
Representation of exposure time and UVI for the generation of vitamin D of 1,000IU and first-degree sunburns for different affected population percentages of a) skin type I, b) skin type II, c) skin type III and d) skin type IV.

Subsequently, cases with 1 %, 50 %, and 97 % of the population with different parts of the body exposed to solar radiation to reach a vitamin D production of 1000 IU were studied. For example, to calculate the time required for 97 % of the population with skin type II that only exposes the face, arms, and hands to the sun (AF = 0.27) to reach 1000 IU of vitamin D and a UVI of 4.5, the first Y must be obtained from equation (12) or (14) or from Finney's PROBIT Table [33], with a value of Y = 6.88. Then, from expression (10) the dose required for this case can be obtained, taking a value of 250 J/m^2^. Finally, with expression (9), the value of time, t_e_ ≈ 10 min, can be obtained. Similarly, the dose necessary for 97 % of the population with type IV skin to reach 1000 IU of vitamin D is 450 J/m^2^. If the UVI is 9 and we assume the same area factor as in the previous case, the time required is approximately 8 min. This result agrees with the value given by Webb and Engelsen for the end of March and June in Boston at local solar noon and a clear day, respectively [27,55]. The studied cases included full body exposure to solar radiation; exposure of the face, hands, arms, and legs; exposure of face, hands, and arms; and exposure of face and hands (Fig. 6(a–d)). Moreover, the results were compared with those of 1 %, 10 %, and 20 % of the population affected by first-degree sunburns.

For all skin types studied, for 97 % of the population to reach the recommended amount of vitamin D when only the face and hands are exposed to solar radiation, 1 % of the population could suffer from first-degree sunburn, as in all cases both lines show close values, especially for high UVI values. The rest of the case studies included less than 1 % of the population affected by first-degree sunburns.

**Fig. 7:**
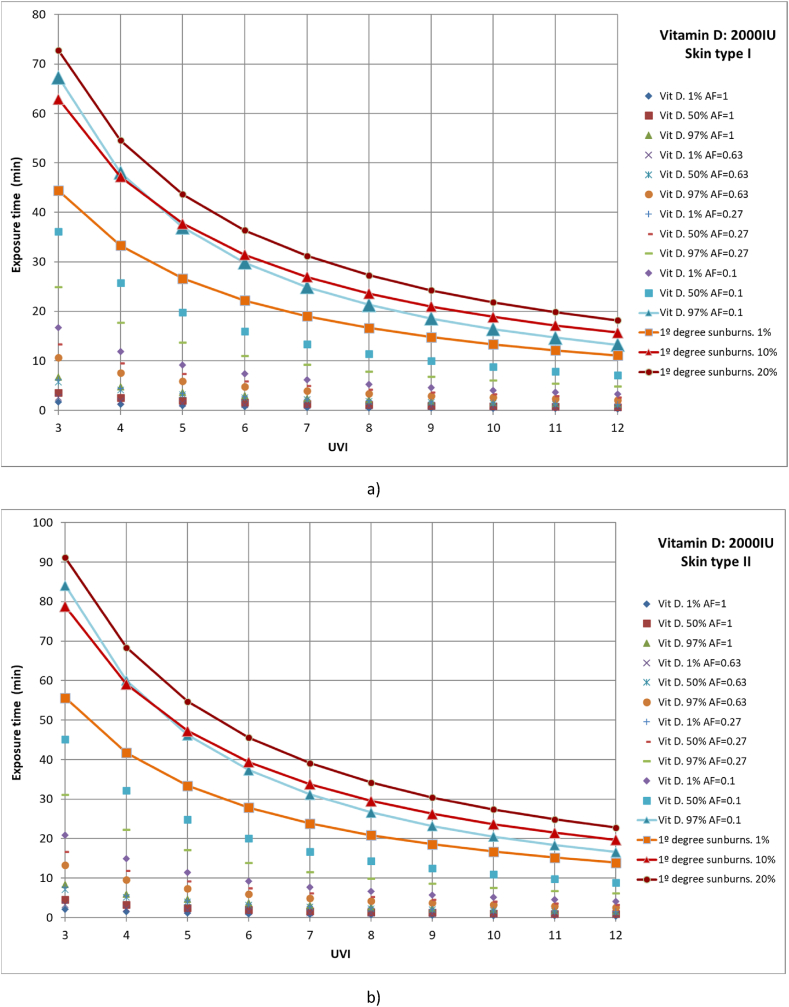

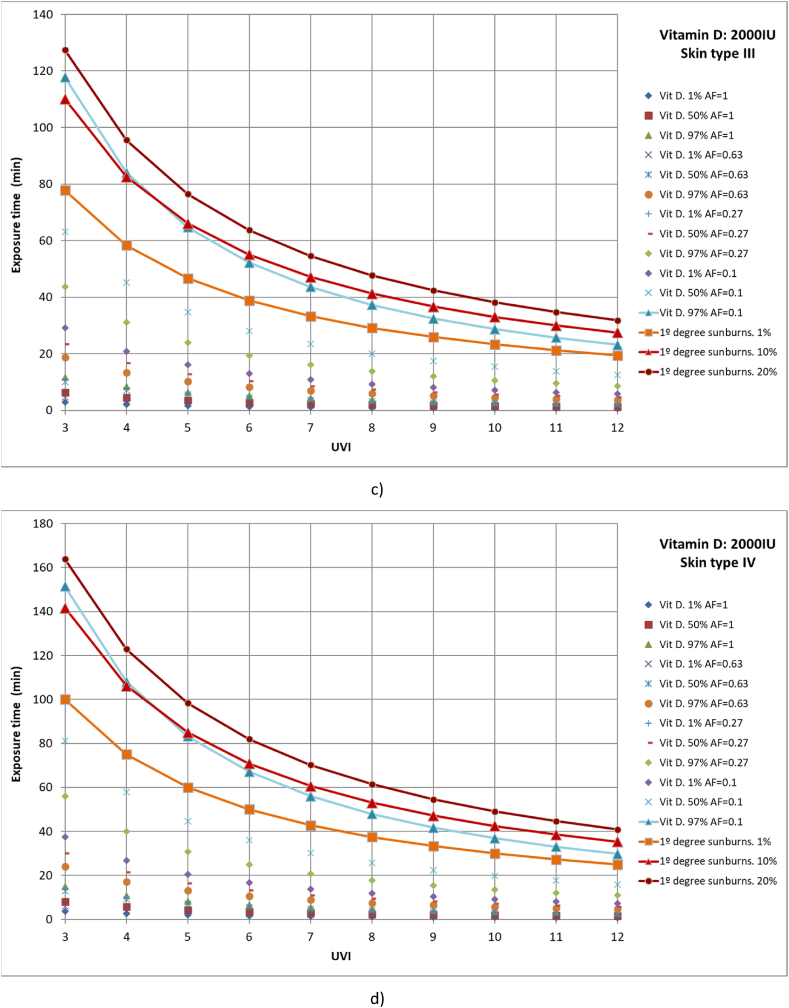
Representation of exposure time and UVI for the generation of vitamin D of 2,000IU and first-degree sunburns for different affected population percentages of a) skin type I, b) skin type II, c) skin type III and d) skin type IV.

Similarly, cases were studied in which 1 %, 50 %, and 97 % of the population had different parts of the body exposed to solar radiation until vitamin D production of 2000 IU was reached. The studied cases include full body exposure to solar radiation; exposure of the face, hands, arms, and legs; exposure of face, hands, and arms; and exposure of face and hands (Fig. 7(a–d)). Moreover, the results are compared with 1 %, 10 % and 20 % of the population affected with first-degree sunburns.

For all skin types studied, for 97 % of the population to reach the recommended amount of vitamin D when only the face and hands are exposed to solar radiation, at least 10 % of the population could suffer from first-degree sunburn, as for all cases both lines are practically coincident, especially for low UVI values. The rest of the case studies are below 1 % of the population affected by first-degree sunburns.

**Fig. 8:**
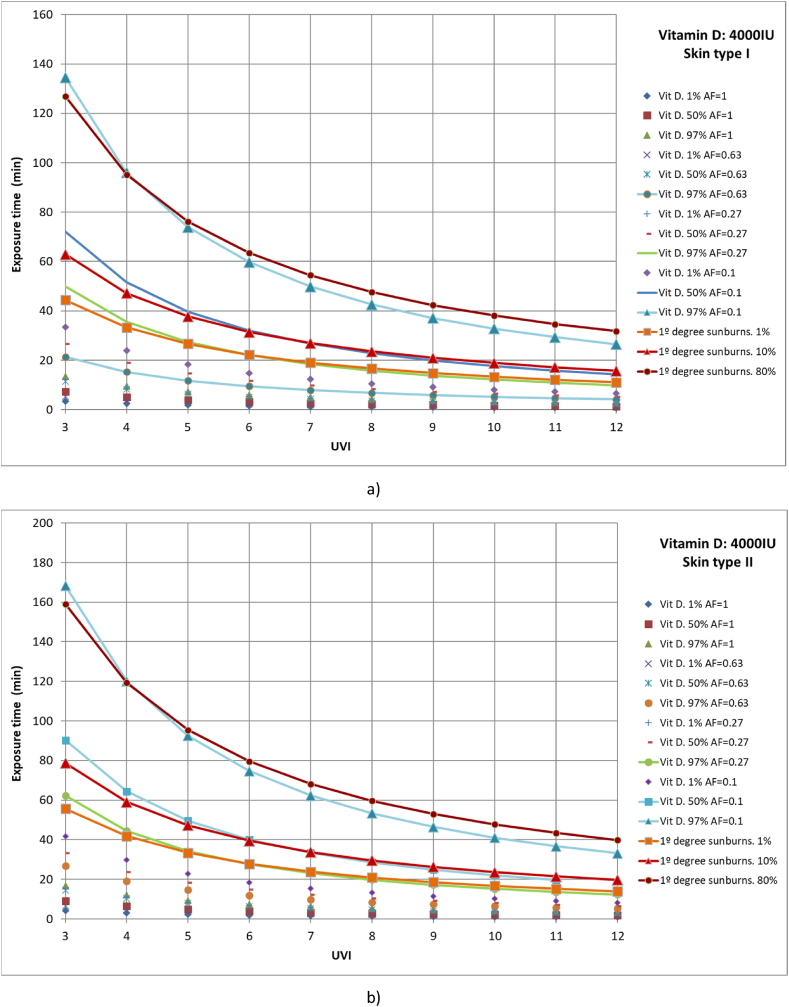

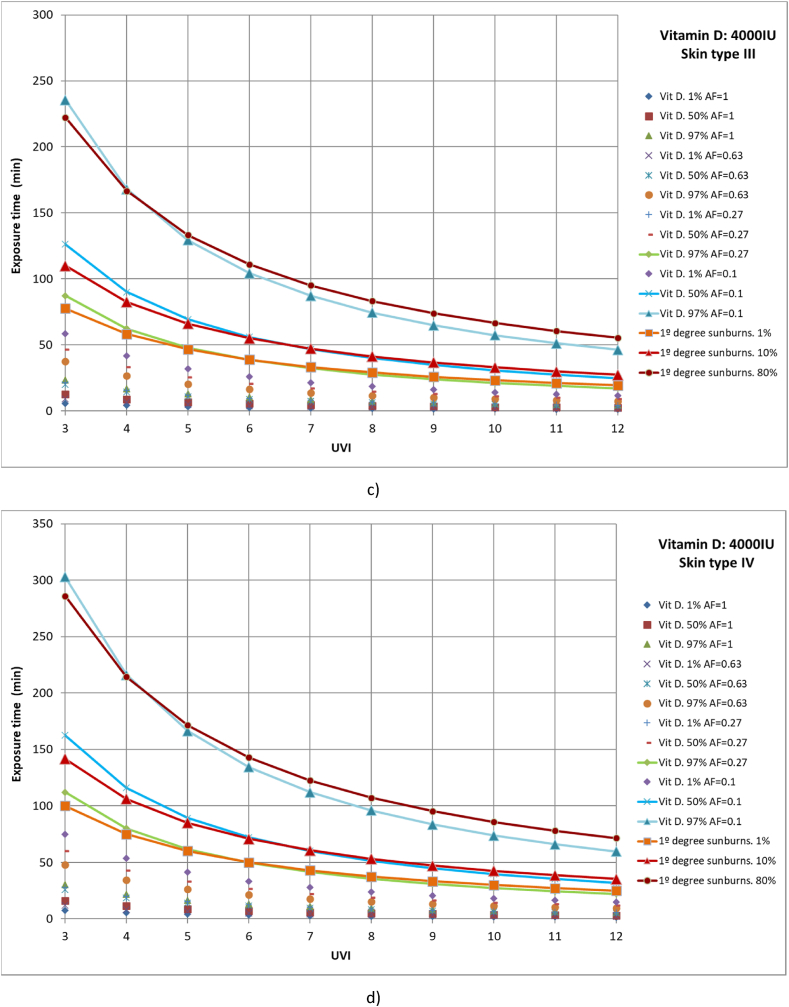
Representation of exposure time and UVI for the generation of vitamin D of 4,000IU and first-degree sunburns for different affected population percentages of a) skin type I, b) skin type II, c) skin type III and d) skin type IV.

Finally, cases were studied in which 1 %, 50 %, and 97 % of the population had different parts of the body exposed to solar radiation until vitamin D production of 4000 IU was reached. The studied cases include full body exposure to solar radiation; exposure of the face, hands, arms, and legs; exposure of face, hands, and arms; and exposure of face and hands (Fig. 8(a–d)). Moreover, the results were compared with those of 1 %, 10 %, and 90 % of the population affected by first-degree sunburns.

For all skin types studied, for 97 % of the population to reach the recommended amount of vitamin D when only the face and hands are exposed to solar radiation, at least 80 % of the population could suffer from first-degree sunburn, as for all cases both lines are practically coincident, especially for low UVI values. On the other hand, both for an area factor (AF) of 0.27 (face, hands and arms exposed to solar radiation) and 97 % of the population reaches the recommended value of vitamin D and for an area factor (AF) of 0.1 (face and hands) and 50 % of the population reaches the recommended value of vitamin D, more than 1 % of the population would suffer first-degree sunburn. In addition, for the second case, values close to 10 % of the population that could suffer first-degree sunburn are presented, since both lines are practically coincident. The rest of the case studies are below 1 % of the population affected by first-degree sunburns.

It should be noted that the use of these types of expressions (PROBIT equations) allows us to calculate the percentage of the population that is affected by different effects caused by the same agent, which is an important educational tool. Thus, the population can know based on their skin type, how long they can expose themselves to solar radiation without suffering first-degree sunburns.

The importance of empirical information and education about the risk of suffering from sunburns can be seen in a study carried out on the Danish population. It is estimated that 4 of every 10 Danes suffer from sunburns per year, making it one of the world's largest populations with incidences of skin cancer. An awareness campaign was launched in 2007–2015, resulting in a reduction of more than 1 % per year in sunburns. This study advocates a future with a predictable, significantly lower incidence of skin cancer [58].

## 4 Final comments and conclusions

In this study, a set of equations was presented that relates the time of exposure to solar radiation, the UVI, and its effects, both positive (synthesis of vitamin D) and negative (sunburn), including the influence of repeated doses. We established the time range in which repeated doses could be applied for each case, both for different IU and skin types. We also included the protective effect of clothes and sunscreens for both the synthesis of vitamin D (where there is a negative effect, as an increase in exposure time is necessary to reach the daily recommended amount of vitamin D) and sunburn (where there is a positive effect because the skin is protected from solar radiation). In addition, the results obtained are in accordance with the empirical evidence. Finally, we propose expressions that relate the percentage of a population that would reach the recommended daily amount of vitamin D and the damage to which that population would be exposed for skin type I to IV.

As a conclusion of this study, it can be highlighted that when a large percentage of a population wants to obtain the daily recommended amount of vitamin D, and by exposing a small portion of the skin to solar radiation, a considerable percentage of the population would suffer first-degree sunburns. More specifically, for a daily amount of 2000 IU or 4000 IU, in all studied skin types, 97 % of the population reaches the recommended amount of vitamin D if they only expose their face and hands to solar radiation, and 10 % or 80 % of the population will suffer first-degree sunburns, respectively. In the same case, for a daily amount of 1000 IU, close to 1 % of the population could suffer first-degree burns. In addition, for 4000 IU, when the face and hands are exposed to solar radiation and 50 % of the population reaches the recommended value of vitamin D, for all skin types, values close to 10 % of the population would suffer first-degree sunburn. This effect is due to the fact that the velocity of vitamin D production is directly related to the area of exposed skin; however, sunburns do not have this relationship, being able to produce localized burns in body parts exposed to solar radiation.

Finally, as a strength of the work carried out, it is worth highlighting that equations are presented that allow calculation of both the percentage of the population that would suffer first-degree burns and the percentage that would reach the recommended amount of vitamin D, and have been corroborated with the information available in the literature. However, it is worth highlighting as a weakness that for the relationships of time necessary to reach a daily amount of 1000 IU of vitamin D at a certain UVI obtained from the bibliography and used in our study to develop the PROBIT expressions, it is understood that practically the entire population would reach this value. Thus, we assumed that 97 % of the population would reach this value, based on the Recommended Dietary Allowance. Another weakness is that the model has been validated with a small sample of data obtained from the work of Webb and Engelsen and McKenzie et al. [22,27]. Finally, it is worth noting as a weakness the assumptions made throughout the article such as the one mentioned above based on the Recommended Dietary Allowance.

## Funding

“This research received no external funding”.

## CRediT authorship contribution statement

**Juan Francisco Sánchez-Pérez:** Writing – review & editing, Writing – original draft, Validation, Methodology, Investigation, Conceptualization. **Begoña Comendador-Jiménez:** Writing – review & editing, Writing – original draft, Methodology, Investigation, Conceptualization. **Enrique Castro:** Writing – review & editing, Writing – original draft, Methodology, Investigation. **Manuel Cánovas:** Writing – review & editing, Writing – original draft, Methodology, Investigation. **Manuel Conesa:** Writing – review & editing, Writing – original draft, Methodology, Investigation.

## Declaration of competing interest

The authors declare that they have no known competing financial interests or personal relationships that could have appeared to influence the work reported in this paper.
